# Supplementary material for: New insights into the plasma and urinary metabolomic signatures of spontaneously hypertensive rats
Source: PLoS One. 2026 Apr 3;21(4):e0344682. doi: 10.1371/journal.pone.0344682 (PMC13048474; doi:10.1371/journal.pone.0344682)

**Supplemental File 1**

**Supplemental Figure 1.** Scores plots corresponding to OPLS-DA models from urine and plasma models


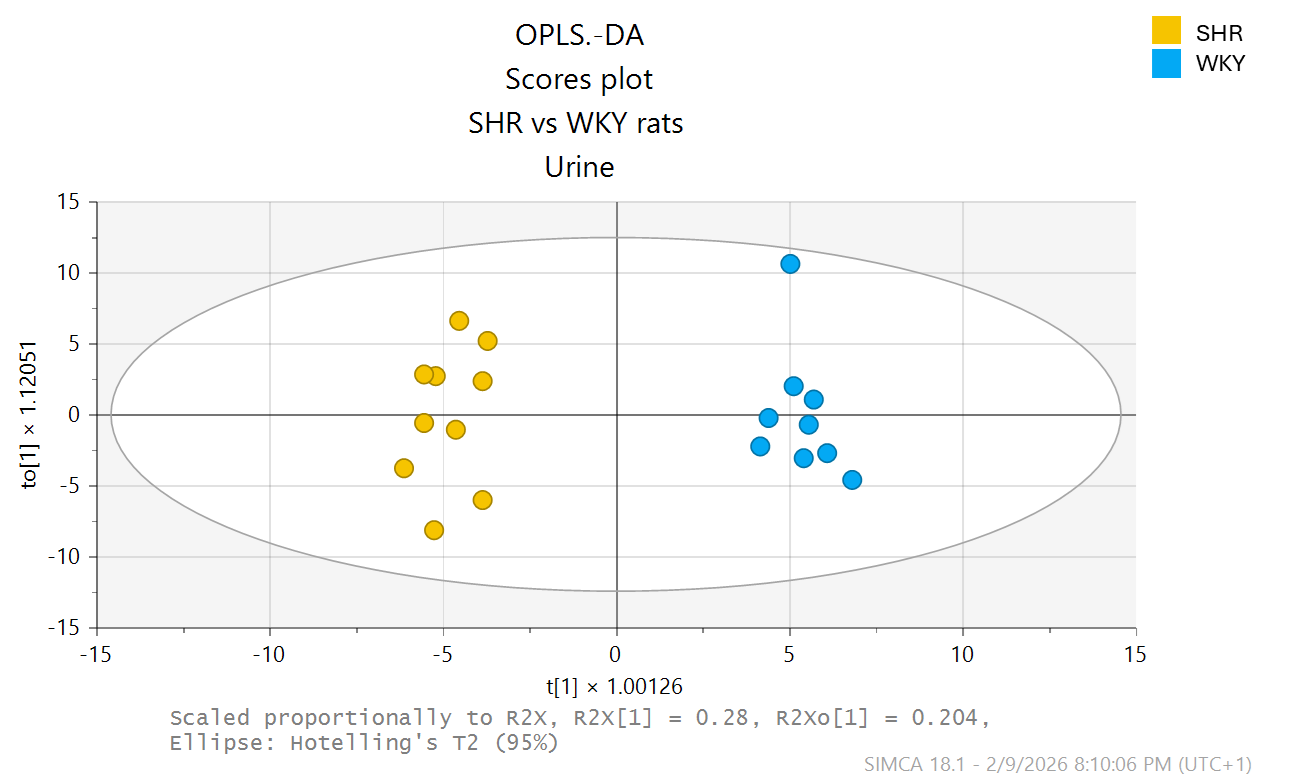


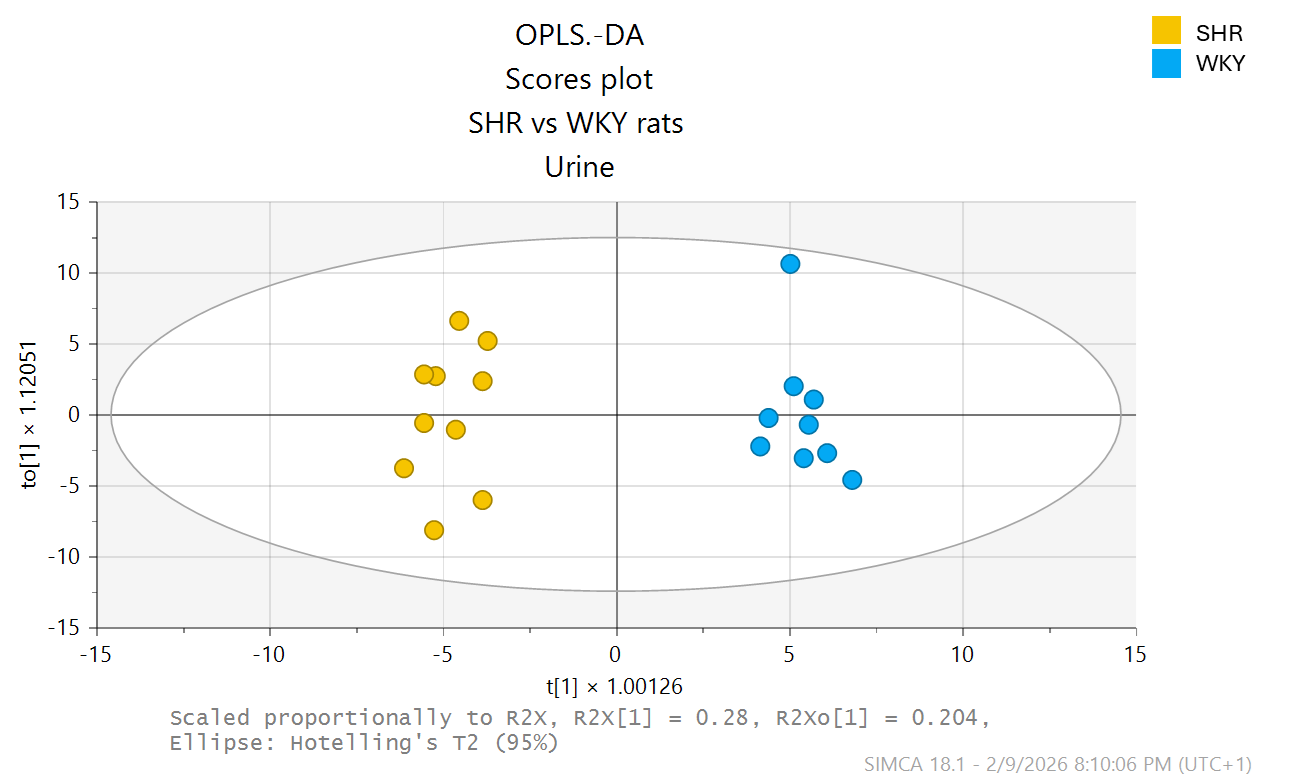


**Supplemental Figure 2.** Permutation plots corresponding to plasma and urine OPLS-DA models


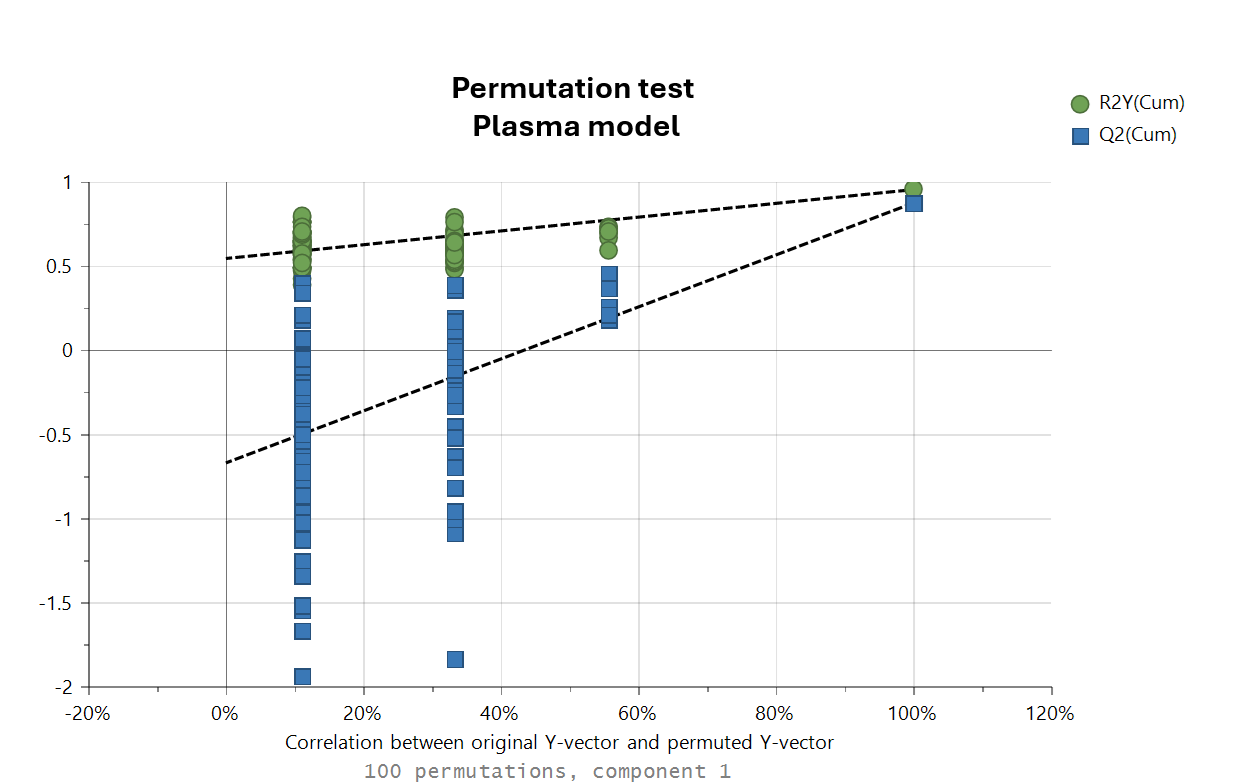


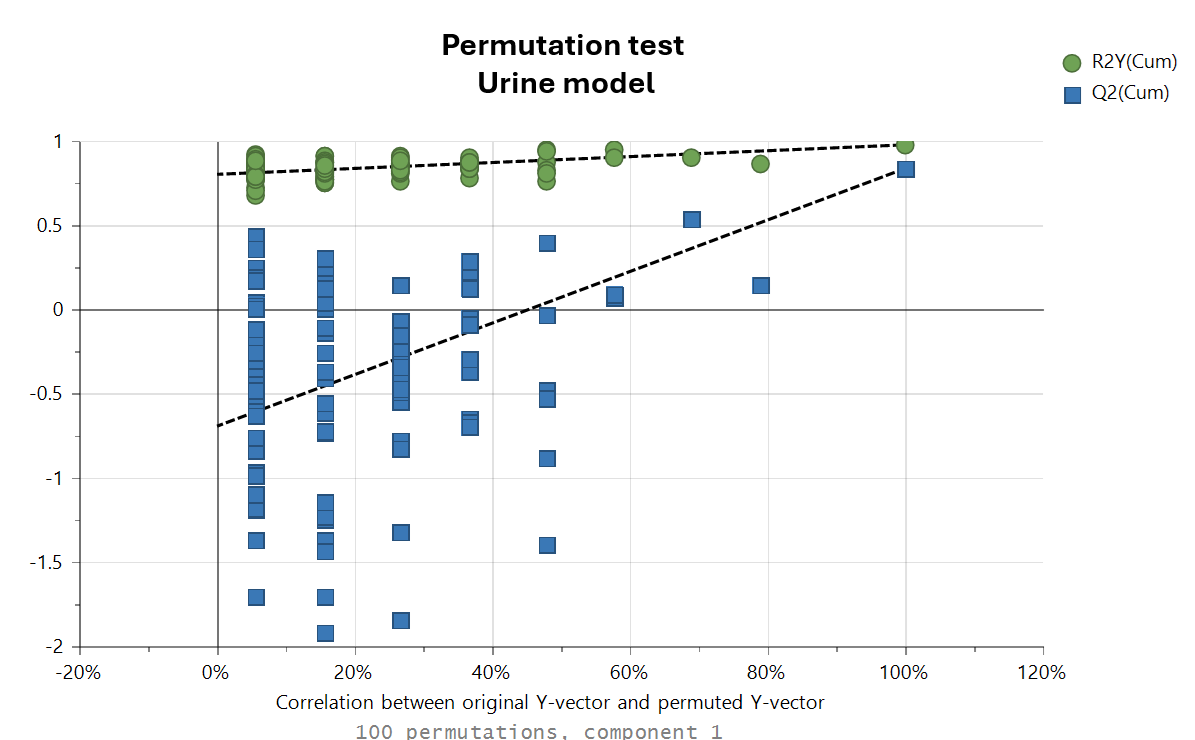


**Supplemental Figure 3.** Representative spectra for discriminant metabolites.

**Compound: RT 5.54 m/z 187.007. p-Cresol sulfate**


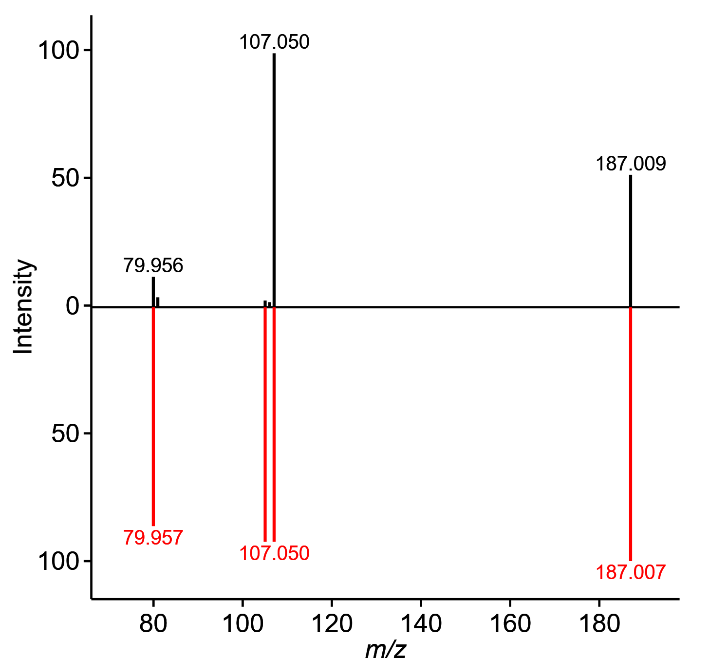


**Compound: RT 12.03 m/z 572.372. LPC (22:4)**


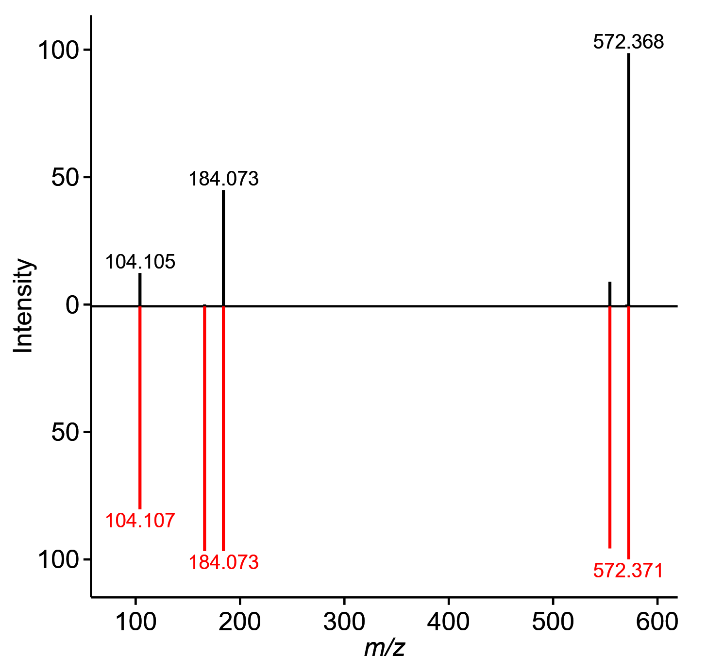


**Compound: RT 11.53 m/z 546.354. LPC (20:3)**


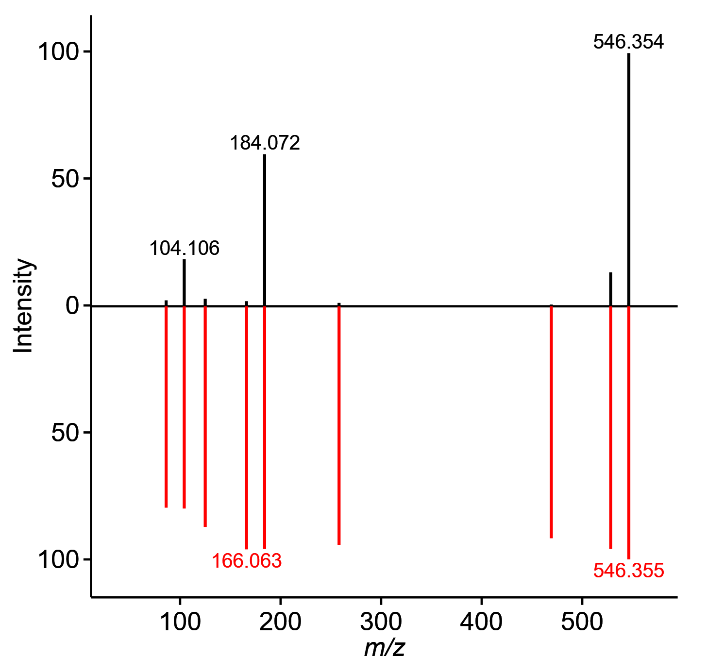


**Compound: RT 10.26 m/z 494.323. LPC (16:1)**


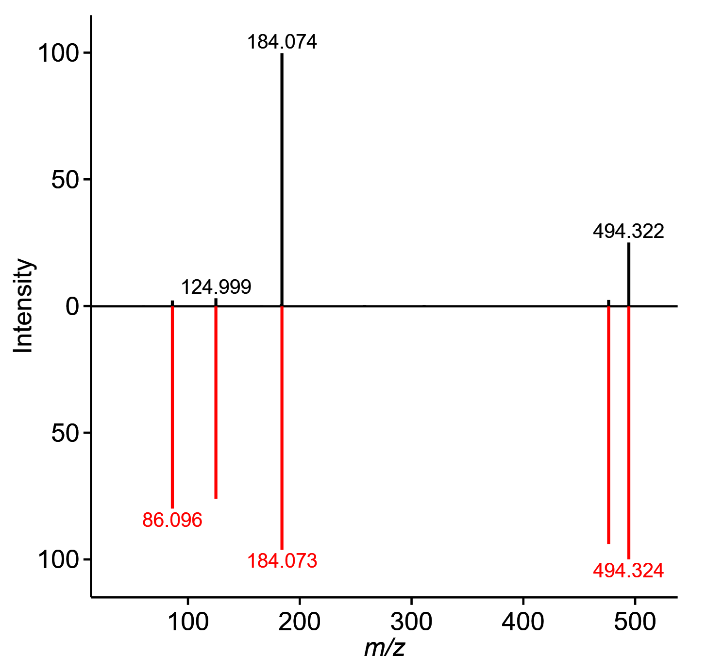


**Compound: RT 10.26 m/z 494.324. LPC (16:1)**


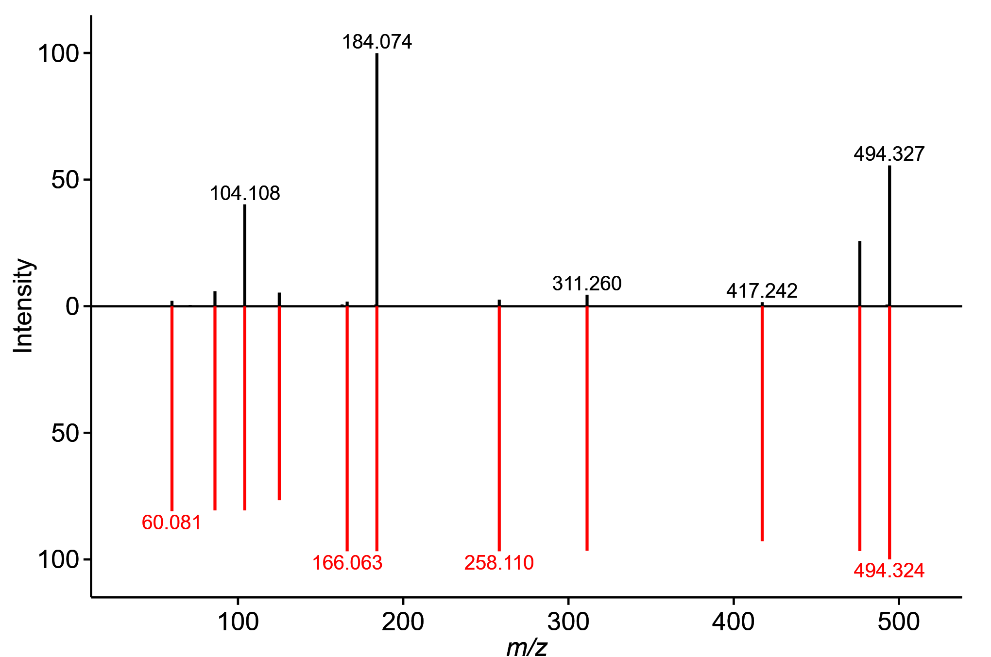


**Compound: RT 9.83 m/z 468.309. LPC (14:0)**


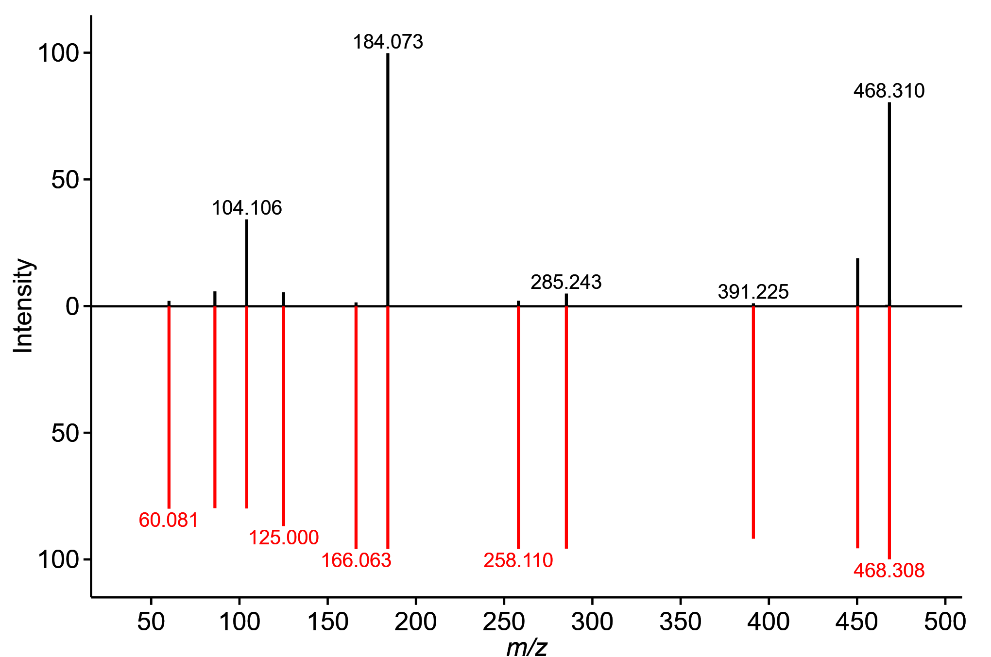


**Compound: RT 8.75 m/z 407.28. Cholic acid**


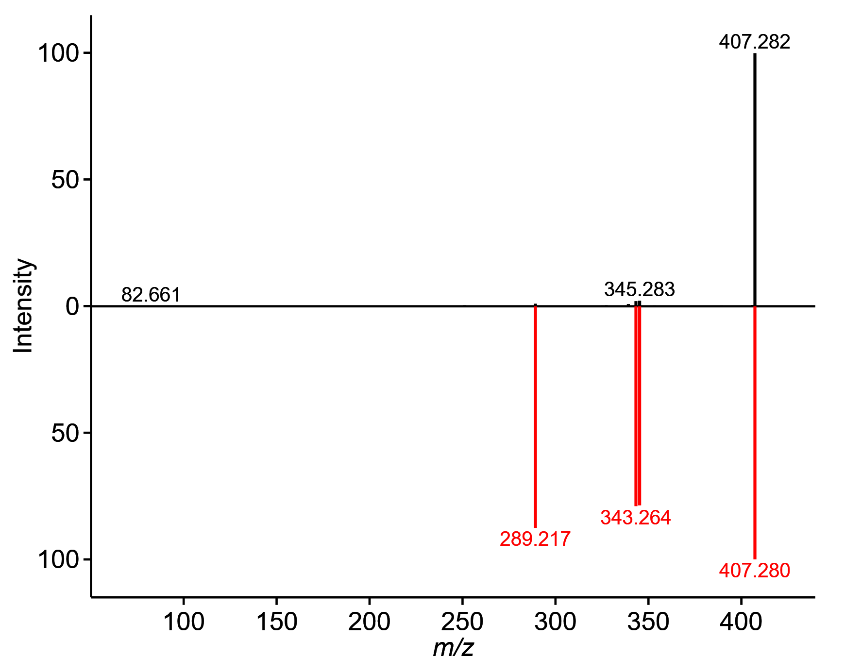


**Compound: RT 10.26 m/z 538.13 LPC (16:1)**


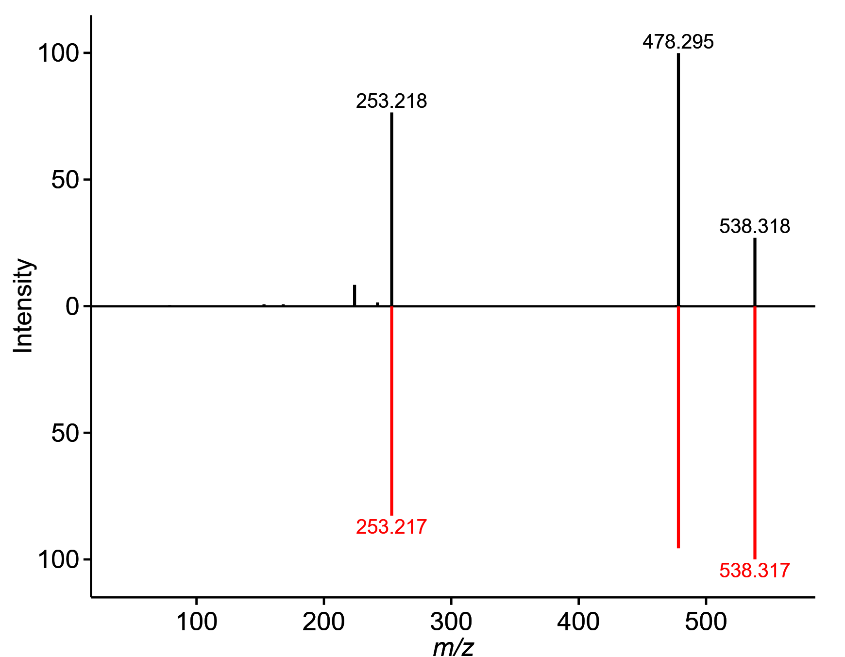


**Compound: RT 1.43 m/z 243.062. Pseudouridine**

**Compound: RT 4.89 m/z 283.0840. P-cresol glucuronide**

**Compound: RT 4.78 m/z 193.0513. Ferulic acid**

**Compound: RT 4.36 m/z 178.0518. Hippuric acid**

**Compound: RT 1.47 m/z 167.0211. Uric acid**

**Compound: RT 1.47 m/z 167.0211. DHPPA**


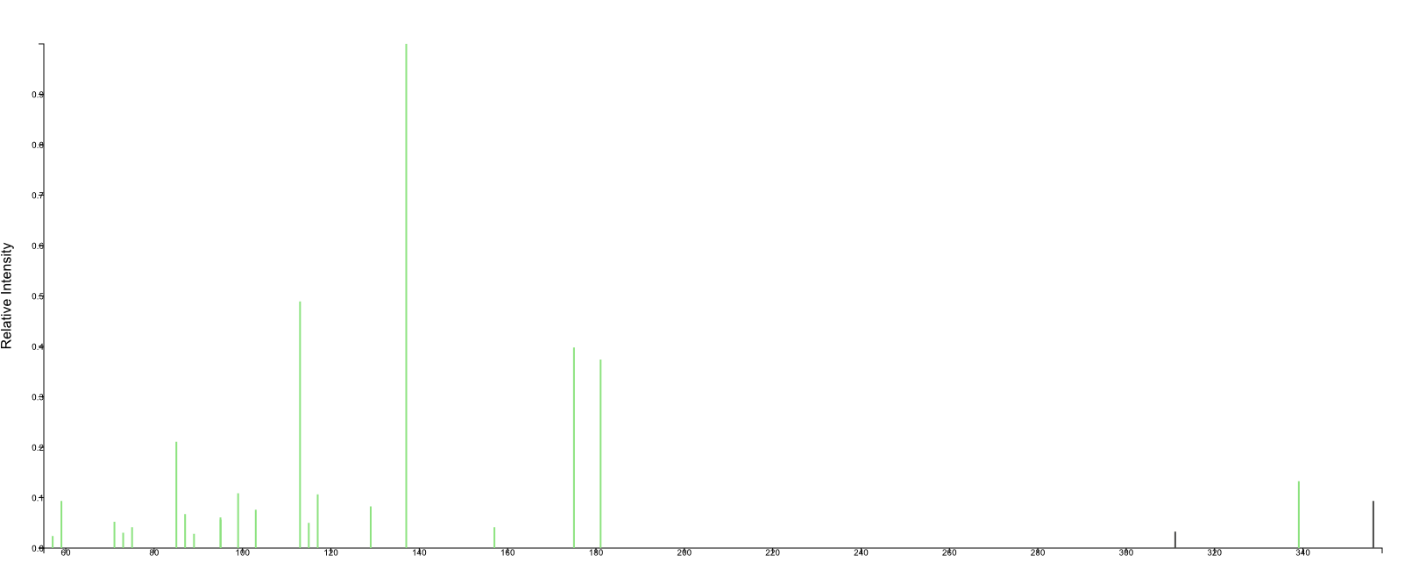


**Compound: RT 11.04 m/z 508.342. LPC 17:1**


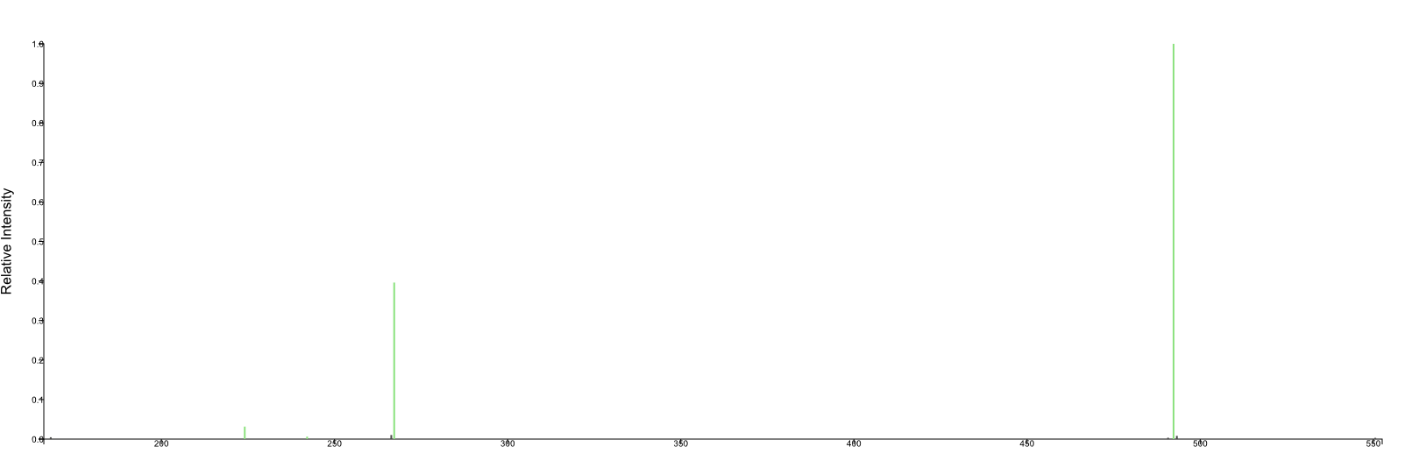


**Compound: RT 11.53 m/z 546.354. LPC 20:3**


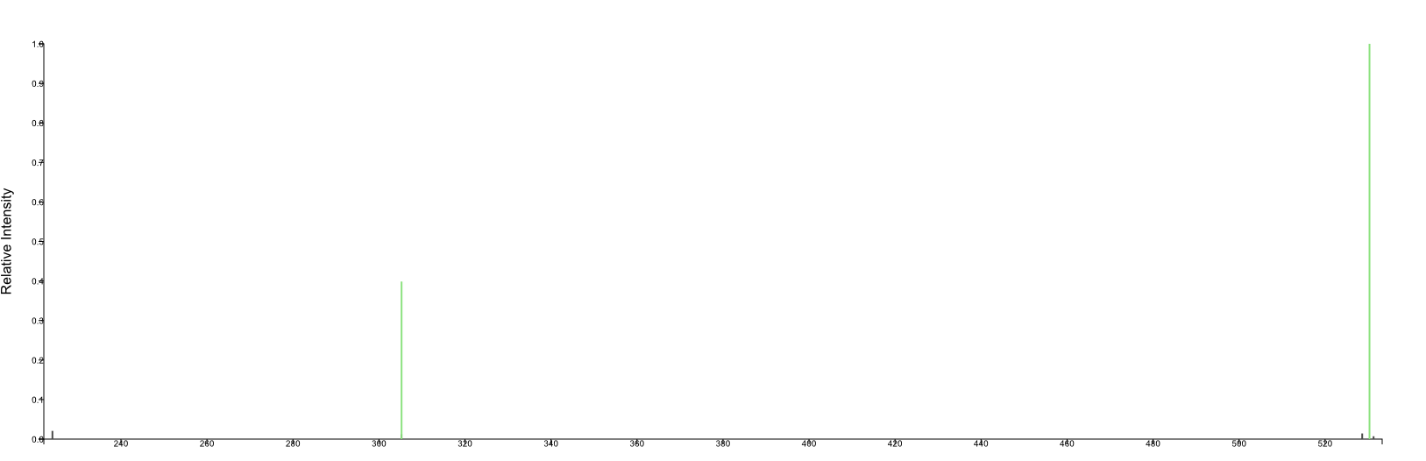


**Compound: RT 1.65 m/z 191.0198. Citric acid**


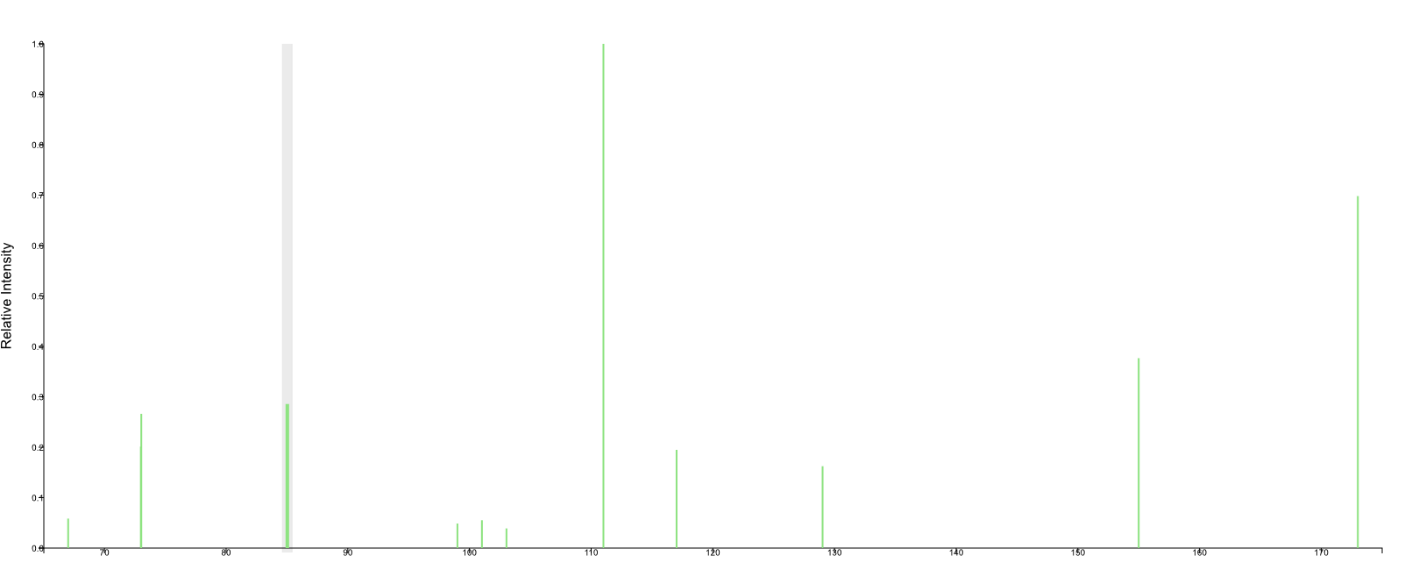
**Compound: RT 5.70 m/z 297.0994. Ethylphenol glucuronide**


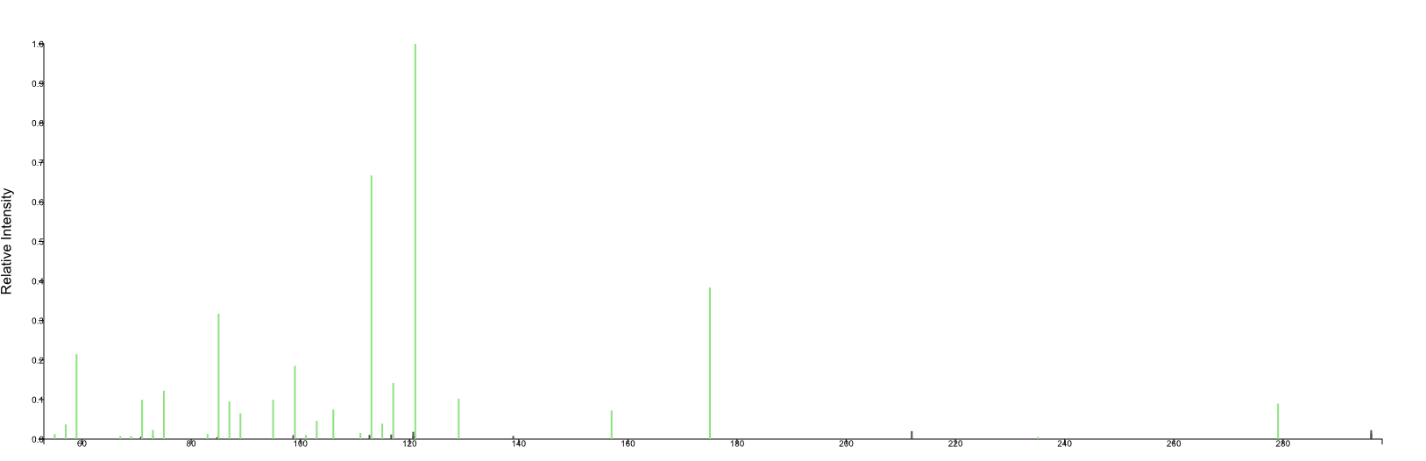


**Compound: RT 5.18 m/z 231.0797. Indole glycine**


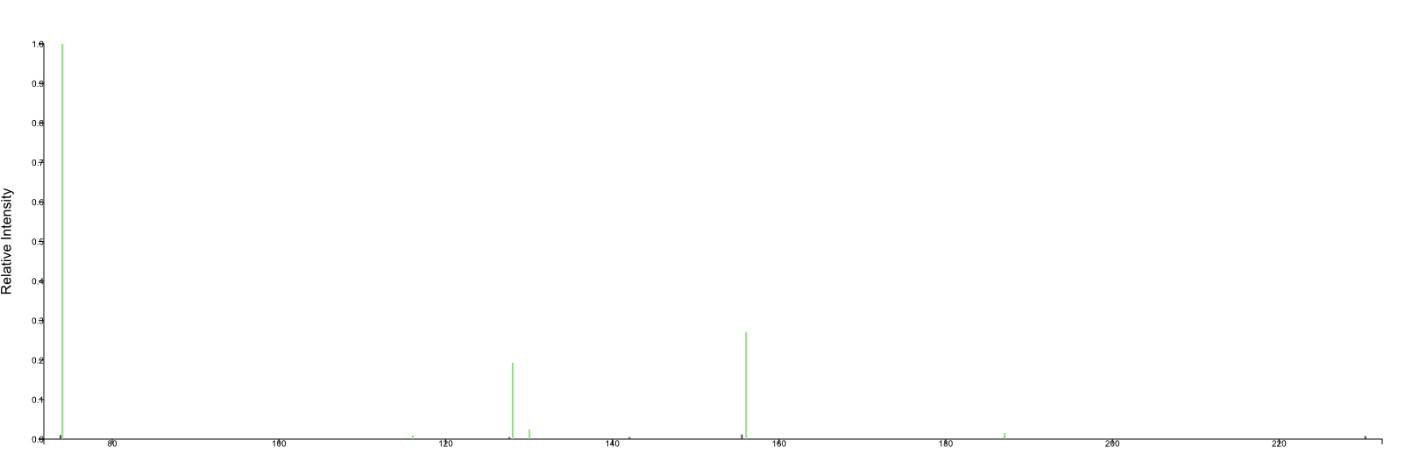


**Compound: RT 5.20 m/z 171.1036. Oxonanoate**


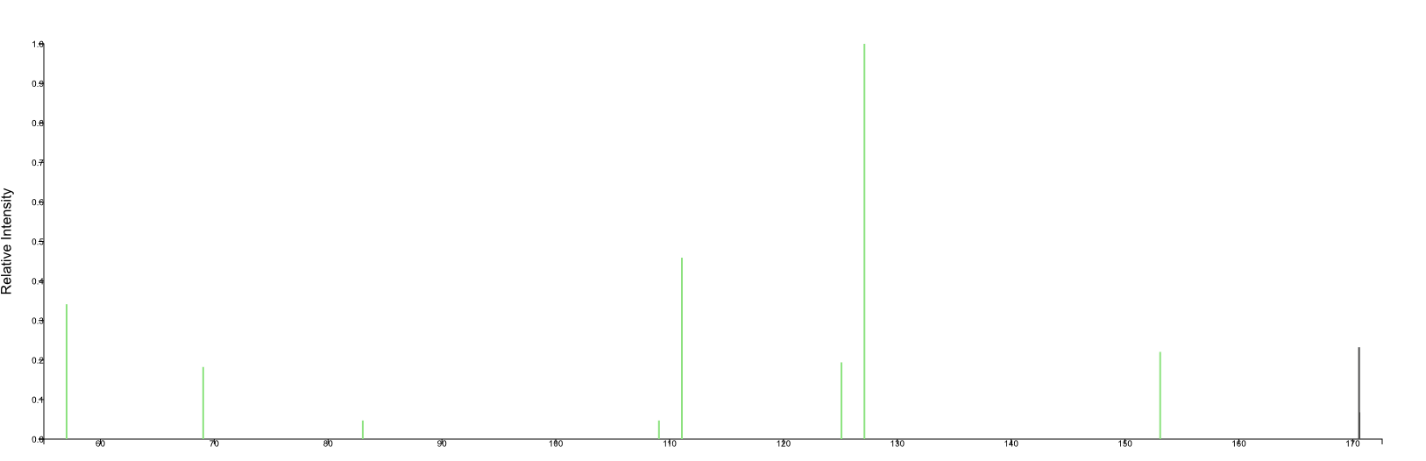

Supplement: S1 File — Spectral comparison between measured MS/MS of the most discriminant metabolites in samples and match in libraries. (DOCX) [file pone.0344682.s001.docx]
