# Supplementary material for: New insights into the plasma and urinary metabolomic signatures of spontaneously hypertensive rats
Source: PLoS One. 2026 Apr 3;21(4):e0344682. doi: 10.1371/journal.pone.0344682 (PMC13048474; doi:10.1371/journal.pone.0344682)
Supplement: Attachment — Submitted filename: Reviewers_response_final.pdf [file pone.0344682.s005.pdf]

6. Review Comments to the Author

Please use the space provided to explain your answers to the questions above. You may also include additional comments for the author, including concerns about dual publication, research ethics, or publication ethics. (Please upload your review as an attachment if it exceeds 20,000 characters)

Reviewer #2: Major Concerns

Unjustified Internal Standard (IS) Selection (Page 8, line 101; Response to Comment 5): The IS mixture used is atypical for a non-targeted metabolomics study of endogenous metabolites. Its composition (mainly pharmaceuticals with only one stable isotope-labeled standard) has poor physicochemical resemblance to the key metabolite classes identified (e.g., bile acids, glycerophospholipids). The authors must provide a detailed justification in the Methods or Discussion for this specific choice, explaining how each component monitors technical variance relevant to their analyte panel. Furthermore, they should critically evaluate and cite the age and context of any literature that employs a similar IS protocol, acknowledging this as a potential limitation for quantitative rigor if modern best practices were not followed.

R: Thank you for the observation. We included internal standards (IS) to monitor instrument stability and to normalize sample-to-sample variation, not for quantitative purposes. To avoid interference with sample signals, we specifically selected IS compounds whose chromatographic peaks do not overlap with signals observed in our samples. This rationale has been validated in our prior studies, where the chosen IS performed as intended without overlapping sample features and without compromising data integrity. Reference 9 has been included in page 8.

Insufficient Metabolite Identification Confidence:
There is a contradiction between the response (stating MS/MS was not run for confirmation) and the data presented in Tables 2 & 3 (listing "Fragments"). The confidence level for all metabolite annotations must be explicitly stated (e.g., following Metabolomics Standards Initiative guidelines). Supporting MS/MS spectra for key annotated metabolites should be provided in the supplementary information.

R: Unfortunately, the response was not correctly formulated. We did not run MS/MS of chemical standards to validate the MS/MS from our analysis. Spectral Data is now attached as Supplemental material and level of confidence is included in the tables.

Deficient Data Presentation and Visualization:

OPLS-DA Plots: The decision to omit score plots is unacceptable. Visual assessment of group separation is essential. The OPLS-DA score plots and corresponding permutation test plots for both plasma and urine models must be provided (Main or Supplementary Figures).

In this context, the plots were omitted, but the model's quality metrics are included. We chose not to present the scores plot because we consider the metrics to be more meaningful; these models often overfit, even with random data, making the plots less informative than the metrics themselves. However, as suggested by the reviewer, the phrase has been rewritten (L198) and the plots are included as supplemental material.

S-Plots (Figure 2): The most discriminating metabolites (variables) should be clearly labeled in the S-plots to align with the data in Tables 2 & 3, enhancing reader interpretation.

R: The information has been attached.

PCA Plot (Figure 1A): The presented plasma PCA plot shows incomplete separation between groups. This should be acknowledged and discussed in the context of the model's performance and the stronger separation suggested by the OPLS-DA parameters. **R: PCA is a non-supervised analysis where no label is assigned to the samples, therefore is a valid approach to explore if clustering is present through an unbiased technique. The separation between groups is clear and complete although there is variation within the groups. The OPLS-DA analysis as a supervised approach is characterized by isolation of any orthogonal variation. In this case, this within group variation is properly handed by the modeling and therefore the group variation is maximized.**

Tables 2 & 3: These tables appear truncated or misformatted in the manuscript file. They must be checked and presented completely and clearly.

R: Tables are now adjusted to a proper format

Specific Editorial and Technical Corrections
Reference Callouts: Several references are listed in the text (e.g., 1, 2, 3) but are not properly cited with superscript numbers in the relevant sentences. All in-text citations must be correctly placed.

R: The references have been checked and modified accordingly.

Formatting Consistency: The manuscript exhibits inconsistent paragraph indentation. Formatting must be unified according to the journal's style guide. Text Errors: Please remove the extra space in line 102.

R: The formatting has been checked and changed accordingly.

Review Comments to the Author

2.To comply with PLOS One submissions requirements, in your Methods section, please provide additional information regarding the experiments involving animals and ensure you have included details on (1) methods of sacrifice, (2) methods of anesthesia and/or analgesia, and (3) efforts to alleviate suffering.

R: AS stated in page 6, lines 73-76. The experiment was performed in accordance with the guidelines set by the European Community Council Directives for the ethical care of animals (86/609/EEC) and were approved by the ethical committee of Laboratory Animals of the University of Granada (Spain; permit number 18/07/2017/099). In addition, we have included the requested additional information in page 7, lines 88-95:

"*The present study was conducted in accordance with the ARRIVE (Animal Research: Reporting of In Vivo Experiments) guidelines. All efforts were made to minimize animal suffering through constant clinical monitoring and environmental enrichment. Chemical restraint was achieved via intraperitoneal (i.p.) administration of Equitensin (2.5 mL/kg). Once a surgical plane of anesthesia was confirmed, blood samples for plasma variable determination were collected via abdominal aortic puncture using a beveled needle for fractional extraction. Finally, rats were euthanized by exsanguination while under deep anesthesia*.**"**
