# Supplementary material for: New insights into the plasma and urinary metabolomic signatures of spontaneously hypertensive rats
Source: PLoS One. 2026 Apr 3;21(4):e0344682. doi: 10.1371/journal.pone.0344682 (PMC13048474; doi:10.1371/journal.pone.0344682)
Supplement: Attachment — Submitted filename: Reviewers comments_PONE-D-25-40750.docx [file pone.0344682.s002.docx]

**Reviewer’s Comments**

**Journal: PLOS One**

**Article: New insights into the plasma and urinary metabolomic signatures of spontaneously**

**hypertensive rats**

**Authors: Huerta et al.**

**Overview**

This work aimed to explore the plasma and urinary non-targeted metabolic profile of 16-week-old spontaneously hypertensive rats (SHR) to identify new metabolomic profiles associated with hypertensive phenotypical characteristics. This study is very important to create better understanding of the pathogenesis of diseases like hypertension and other cardiovascular diseases and their complications. Additionally, it will also identify biomarkers for these conditions as well as associated metabolic mechanisms and pathways by which they modulate these conditions. The study therefore, hoped to offer alternative approaches for treatment of hypertension and cardiovascular diseases. The concept was well thought out and the various analyses were clearly designed to address the objectives of the study. The results obtained and reasons adduced for the reported effects and conclusions drawn are succinct to support the mechanisms. However, there were so many flaws in the study in its current form that should be resolved to make it publishable.

**Major comments**

1. The authors did not state the age at which the animals were purchased? Were they purchased at 5 weeks of age and kept until 16 weeks of age before termination and sample collection? Or, were they purchased at 16 weeks which was the time of termination and sample collection?
2. How long was the “SBP was monitored to evaluate the state of the disease?”
3. Was SBP measurement done in conscious or unconscious animals? If in conscious state, what measures were taken to restrain the animals so that BP readings are not affected by their unrestrained movements? If in unconscious state, what was the procedure for rendering the animals unconscious? The method was not detailed enough to give the reader a clearer picture for replication.
4. How ideal is the *Mummichog* software for biomarker identification since it only identifies significantly enriched metabolic pathways to create hypotheses by bypassing the need to fully identify every single metabolite? Without fully identifying individual metabolites in the system, it will be difficult to have a clear picture of metabolites that may be significantly changing and could be considered biomarkers for the disease or condition.
5. For the plasma and urine metabolomics samples preparation, were internal standards used, and if so, what was the internal standard and at what concentration/sample?
6. In the results section (3.1), the authors discussed the heart weight, left ventricular weight, water intake, diuresis, and heart rates and other organ weights. These were not discussed in the methodology section previously to give an idea of what was done in the various methods, especially with regards to organ collection.
7. Page 11, last paragraph, “The supervised multivariate analysis OPLS-DA indicated a strong separation between the two groups (data not shown).” Why would the data for such an important information not be shown to give readers an idea of the separation between the 2 groups?
8. Page 24, Table 1; why the middle line between heart rate and tibia length?
9. Page 25, Table 2; the authors need to create a column to shown which of the metabolites were either upregulated or down-regulated.
10. Page 26, Table 3; the authors need to create a column to shown which of the metabolites were either upregulated or down-regulated.
11. I am not sure if figure 1 was for this particular study, as it resembles a clinical study more than the animal study.
12. The figures are not properly aligned with the titles. Check that they represent what they claimed to be.
13. Notable pathways and mechanisms for hypertension especially oxidative stress and inflammation were not reported by this study, suggesting a limitation in the *Mummichog* software used and therefore, the need for more high throughput software to validate the outcomes.

**Minor comments**

1. There are some typographical errors throughout the texts that needs revisions. Since they manuscript lacked line numbers, it was difficult outlining specific errors.
