# Supplementary material for: New insights into the plasma and urinary metabolomic signatures of spontaneously hypertensive rats
Source: PLoS One. 2026 Apr 3;21(4):e0344682. doi: 10.1371/journal.pone.0344682 (PMC13048474; doi:10.1371/journal.pone.0344682)
Supplement: Attachment — Submitted filename: Reviewers comments_PONE-D-25-40750_final.docx [file pone.0344682.s004.docx]

**Reviewer’s Comments**

**Journal: PLOS One**

**Article: New insights into the plasma and urinary metabolomic signatures of spontaneously**

**hypertensive rats**

**Authors: Huerta et al.**

**Overview**

This work aimed to explore the plasma and urinary non-targeted metabolic profile of 16-week-old spontaneously hypertensive rats (SHR) to identify new metabolomic profiles associated with hypertensive phenotypical characteristics. This study is very important to create better understanding of the pathogenesis of diseases like hypertension and other cardiovascular diseases and their complications. Additionally, it will also identify biomarkers for these conditions as well as associated metabolic mechanisms and pathways by which they modulate these conditions. The study therefore, hoped to offer alternative approaches for treatment of hypertension and cardiovascular diseases. The concept was well thought out and the various analyses were clearly designed to address the objectives of the study. The results obtained and reasons adduced for the reported effects and conclusions drawn are succinct to support the mechanisms. However, there were so many flaws in the study in its current form that should be resolved to make it publishable.

**Major comments**

1. The authors did not state the age at which the animals were purchased? Were they purchased at 5 weeks of age and kept until 16 weeks of age before termination and sample collection? Or, were they purchased at 16 weeks which was the time of termination and sample collection?

**R: Thank you for your comments. Rats were purchased at 8 weeks of age and kept until 16 weeks of age before termination and sample collection. We have clarified this issue in Page 6, line 64.**

2. How long was the “SBP was monitored to evaluate the state of the disease?”

**R: Thank you for your comments, SBP was monitored to check the evolution of the disease. We have clarified this issue in Page 7, line 78-84.**

3. Was SBP measurement done in conscious or unconscious animals? If in conscious state, what measures were taken to restrain the animals so that BP readings are not affected by their unrestrained movements? If in unconscious state, what was the procedure for rendering the animals unconscious? The method was not detailed enough to give the reader a clearer picture for replication.

**R: Systolic blood pressure (SBP), diastolic blood pressure (DBP), and heart rate (HR) are measured in conscious rats using a non-invasive tail-cuff plethysmography system (LE 5001 Pressure Meter, Letica SA, Barcelona, Spain). Animals are first habituated to the procedure on several days prior to the experiment to minimize stress-related artifacts. On the day of measurement, rats are placed in individual restrainers that allow immobilization without the use of anesthesia. The tail is gently extended through the rear opening of the restrainer. This information has been included in Page 7, line 78-84.**

4. How ideal is the *Mummichog* software for biomarker identification since it only identifies significantly enriched metabolic pathways to create hypotheses by bypassing the need to fully identify every single metabolite? Without fully identifying individual metabolites in the system, it will be difficult to have a clear picture of metabolites that may be significantly changing and could be considered biomarkers for the disease or condition.

**R: The results of mummichog are very relevant to provide an insight into the pathways that might be altered. We used this approach for double crossing with the univariate/multivariate analysis. As we had some potential annotations but we were not able to run MS/MS experiments to confirm the identity, we used the combined information to increase the confidence in our annotations.**

5. For the plasma and urine metabolomics samples preparation, were internal standards used, and if so, what was the internal standard and at what concentration/sample?

**R: This information was included in page 8 as follows: *“****Five microliters of internal standard (IS) consisting of a mixture of roxithromycin 3 ppm, tryptophan-D5 5 ppm, hydroxydiclofenac 5 ppm, warfarin 3 ppm, ibuprofen 5 ppm, and bisphenol 3 ppm , were added prior the extraction*”.

6. In the results section (3.1), the authors discussed the heart weight, left ventricular weight, water intake, diuresis, and heart rates and other organ weights. These were not discussed in the methodology section previously to give an idea of what was done in the various methods, especially with regards to organ collection.

**R: Methodology details has been included:**

**“Food and water intake were monitored daily by weighing the remaining food or by measuring the remaining volume of water, respectively, allowing the calculation of daily food and water consumption.” (in page 7, lines 85-88).**

**“In addition, after euthanasia, necropsy was performed and the heart, kidneys, liver, and tibia were collected and their absolute weights recorded to evaluate animal development and organ-specific involvement.” (in page 7, lines 90-93).**

7. Page 11, last paragraph, “The supervised multivariate analysis OPLS-DA indicated a strong separation between the two groups (data not shown).” Why would the data for such an important information not be shown to give readers an idea of the separation between the 2 groups?

**R: In this context, the plots are omitted, but the model's quality metrics are included. We chose not to present the scores plot because we consider the metrics to be more meaningful; these models often overfit, even with random data, making the plots less informative than the metrics themselves. The phrase has been rewritten (L198)**

8. Page 24, Table 1; why the middle line between heart rate and tibia length?

**R: Thank you for the comment. This was an error that has been eliminated.**

9. Page 25, Table 2; the authors need to create a column to shown which of the metabolites were either upregulated or down-regulated.

**R: The column is now included to facilitate the interpretation of the results.**

10. Page 26, Table 3; the authors need to create a column to shown which of the metabolites were either upregulated or down-regulated.

**R: The column is now included to facilitate the interpretation of the results.**

11. I am not sure if figure 1 was for this particular study, as it resembles a clinical study more than the animal study.

**R: The correct figure is now attached.**

12. The figures are not properly aligned with the titles. Check that they represent what they claimed to be.

**R: The correct figure is now attached.**

13. Notable pathways and mechanisms for hypertension especially oxidative stress and inflammation were not reported by this study, suggesting a limitation in the *Mummichog* software used and therefore, the need for more high throughput software to validate the outcomes.

**R: The use of Mummichog in our study is based on its ability to infer metabolic pathway enrichment directly from global metabolomics data without requiring prior identification of all metabolites, which is particularly suitable for exploratory analyses based on high-resolution LC-MS. The absence of pathways classically associated with hypertension, such as oxidative stress or inflammation, does not necessarily imply that these biological processes are not involved, but may reflect inherent limitations in the coverage of metabolites in reference databases, the representation of these pathways, and the statistical criteria for enrichment. In this context, pathways with a small number of annotated metabolites or insufficiently represented m/z signals may not reach statistical significance, even when the underlying processes are biologically active. Therefore, this limitation is not unique to Mummichog, but common to enrichment-based pathway analysis approaches in untargeted metabolomics studies. We agree with the reviewer that the application of complementary tools and more in-depth approaches, including methods based on identified metabolites and cross-validation with alternative software, could strengthen the biological interpretation and will be considered in future studies.**

**Minor comments**

1. There are some typographical errors throughout the texts that needs revisions. Since they manuscript lacked line numbers, it was difficult outlining specific errors.

**R: The manuscript has been carefully revised, and several corrections were made across it.**
